# CeO_2_-Based Two-Dimensional Layered Nanocomposites Derived from a Metal–Organic Framework for Selective Electrochemical Dopamine Sensors

**DOI:** 10.3390/s20174880

**Published:** 2020-08-28

**Authors:** Chengjie Ge, Rajendran Ramachandran, Fei Wang

**Affiliations:** 1 School of Microelectronics, Southern University of Science and Technology, Shenzhen 518055, China; 11712522@mail.sustech.edu.cn (C.G.); ramachandran@sustech.edu.cn (R.R.); 2 SUSTech Academy for Advanced Interdisciplinary Studies, Southern University of Science and Technology, Shenzhen 518055, China; 3 Engineering Research Center of Integrated Circuits for Next-Generation Communications, Ministry of Education, Shenzhen 518055, China

**Keywords:** differential pulse voltammetry, dopamine, CeO_2_, siloxene

## Abstract

In this work, we demonstrate the incorporation of two-dimensional (2D) layered materials into a metal–organic framework (MOF) derived from one-dimensional (1D) cerium oxide (CeO_2_) for the electrochemical detection of dopamine. Ce-MOF was employed as a sacrificial template for preparing CeO_2_ with 2D materials by the pyrolysis process. The influence of the pyrolysis temperature was studied to achieve a better crystal structure of CeO_2_. Siloxene improved the dopamine sensing performance of CeO_2_ compared with graphitic carbon nitride (g-C_3_N_4_) due to the basal plane surface oxygen and hydroxyl groups of 2D siloxene. Under optimal conditions, the fabricated CeO_2_/siloxene electrode exhibited a detection limit of 0.292 μM, with a linear range from 0.292 μM to 7.8 μM. This work provides a novel scheme for designing the CeO_2_ material with siloxene for excellent dopamine sensors, which could be extended towards other biosensing applications.

## 1. Introduction

Dopamine (DA) is one of the most abundant catecholamine neurotransmitters, playing a crucial role in the human brain [1,2]. Abnormal dopamine content causes a few diseases, like brain aging, Parkinson’s syndrome, and schizophrenia. Thus, the detection of DA molecules in the central nervous system is becoming a necessary and significant task in the biosensor field [3,4]. Till now, many methods have been used to detect dopamine molecules, such as spectrophotometry, titrimetry, the chemiluminescence method, and the electrochemical method. Among these, the electrochemical detection method offers high sensitivity, low cost, and excellent selectivity. Hence, the electrochemical detection of DA has been considered to be effective for practical usage [5,6,7,8,9,10].

On the other hand, metal–organic frameworks (MOFs) and their derived composites have drawn much interest in various applications, such as energy storage, conversion, catalysis, separation, and gas sensors, thanks to their superior material properties like a large surface area, a tunable structure, and high thermal and chemical stability [11,12,13,14,15]. Additionally, an MOF has been employed as a flexible template to prepare porous metal oxides/sulfides and carbon materials using the direct pyrolysis process [16,17,18,19]. Up to now, various metal oxides such as Co_3_O_4_, CuO, cerium oxide (CeO_2_), and NiO have been synthesized using MOFs as their sacrificial template and applied in various electrochemical applications, such as supercapacitors, batteries, and biosensors [20,21,22,23].

Among them, CeO_2_ is a rare earth metal oxide that has attracted much interest in biomolecules detection due to its facile oxygen vacancy formation properties, excellent biocompatibility, and natural abundance. Furthermore, the stable redox behavior between Ce^3+^ and Ce^4+^ enhances the electrocatalytic properties of CeO_2_. Nevertheless, attaining highly electrocatalytic and selective CeO_2_ for DA detection is a challenging task, as a result of its poor electrical conductivity [24,25,26]. Therefore, for high sensitivity and selectivity it could be an effective strategy to combine two-dimensional (2D) conductive materials with CeO_2_.

Emerging two-dimensional materials such as siloxene and graphitic carbon nitride (g-C_3_N_4_) have recently been used as excellent substrates for the electrochemical sensor field because of their high surface area, excellent electrical conductivity, and better redox abilities [27,28,29,30,31]. A large number of experiments have been performed on g-C_3_N_4_ and other two-dimensional materials combined with MOFs and metal oxides for electrochemical sensors. The 2D conductive materials can increase the rate of electron transport, resulting in high electrocatalytic activity [32,33,34]. Siloxene, a type of low-buckled structure, has been explored as an excellent candidate for the dopamine sensor, and the reported detection limit was 0.327 μM [27].

Taking into consideration the above factors, we successfully synthesized the 2D siloxene and g-C_3_N_4_ with CeO_2_ and studied their electrocatalytic properties for DA detection. Although a few reports are available on MOF-derived CeO_2_ for electrochemical applications, there has been no reported work on the combination of CeO_2_ with g-C_3_N_4_ and siloxene, derived from a Ce-MOF for electrochemical dopamine sensors. This paper demonstrates the simple wet chemical and pyrolysis routes for the preparation of CeO_2_/siloxene and CeO_2_/g-C_3_N_4_ composites from Ce-MOF materials and investigates their electrochemical sensing ability. Combining 2D siloxene and g-C_3_N_4_ can significantly enhance the electroactive sites of CeO_2_ for DA detection. As a result of the synergistic effect of CeO_2_ and g-C_3_N_4_/siloxene, the composites exhibited a better performance towards DA detection. Interestingly, CeO_2_/siloxene showed higher detection performance than CeO_2_/g-C_3_N_4,_ with a detection limit of 0.292 μM. The strong coordination modes between the Ce atom and siloxene oxygen functional groups in the CeO_2_/siloxene composite are beneficial for improving the DA oxidation performance with good selectivity and stability, which can be applied in future electrochemical sensor applications.

## 2. Materials and Methods

### 2.1. Materials

Cerium (II) nitrate hexahydrate (Ce(NO_3_)_2_·6H_2_O), 1,3,5-tricarboxylic acid (H_3_BTC), urea (CH_4_N_2_O), and hydrochloric acid (HCl, 37% assay) were purchased from Sigma–Aldrich. Calcium disilicide (CaSi_2_) was purchased from Alfa Asear, China. All of the solutions were prepared using Milli-Q water (pH 7.2).

### 2.2. Preparation of Ce-MOF, Ce-MOF/Siloxene, and Ce-MOF/g-C_3_N_4_ Composites

Siloxene and g-C_3_N_4_ samples were synthesized based on our previous reports [27,35]. In line with our previous report, the Ce-MOF-based composites were synthesized by a simple wet chemical route [36]. Briefly, 0.454 g of Ce(NO_3_)_2_·6H_2_O was added into a 40 mL of water and ethanol solution (3:1 volume ratio) and stirred for 10 min (mixture I). Meanwhile, 0.494 g of H_3_BTC was added into another 40 mL 25% ethanol solution and stirred for 15 min. (mixture II). Afterwards, mixture I was added drop by drop into mixture II with continuous stirring. Finally, the solid white powder was washed and centrifuged several times with ethanol and water, then dried in the vacuum oven at 80 °C for 12 h. The product was named Ce-MOF. Ce-MOF/siloxene and Ce-MOF/g-C_3_N_4_ were also synthesized by a similar procedure as the abovementioned, with the addition of 20 mg of siloxene and g-C_3_N_4_ into the H_3_BTC solution.

### 2.3. Preparation of CeO_2_, CeO_2_/Siloxene and CeO_2_/g-C_3_N_4_

**Scheme 1:**
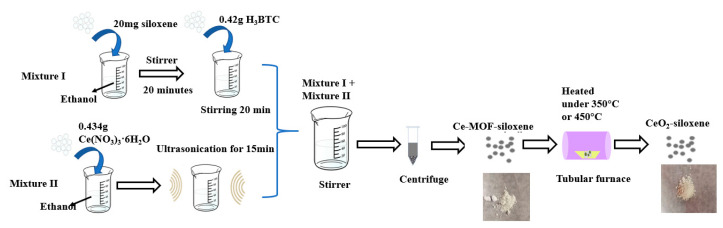
Schematic diagram of cerium oxide (CeO_2_)/siloxene composite preparation.

The bare CeO_2_ and composites with siloxene and g-C_3_N_4_ were prepared by the pyrolysis of Ce-MOF, Ce-MOF/siloxene, and Ce-MOF/g-C_3_N_4_, respectively. The pyrolysis temperature was set to 350 and 450 °C for the three samples. The process of CeO_2_/siloxene composite preparation is illustrated in Scheme 1.

### 2.4. Materials Characterization

The Rigaku Smartlab diffractometer collected the powder X-ray diffraction (XRD) pattern of the prepared samples. Cu-Kα radiation (λ = 1.540 Å) was utilized to characterize the samples with the 2θ interval from 6 to 70°. A scanning electron microscope (SEM, Zeiss Merlin, Jena, Germany) and the high-resolution transmission electron microscope (TEM, Tecnai F30, FEI Ltd., Hillsboro, OR, USA) were used to identify the samples’ structural morphology. The porosity and surface area of the samples was characterized by the Brunauer–Emmett–Teller (BET, ASAP2020, Duluth, GA, USA) instrument.

### 2.5. Preparation of Modified Electrodes for Dopamine Detections and Electrochemical Analysis of Detections

**Scheme 2:**
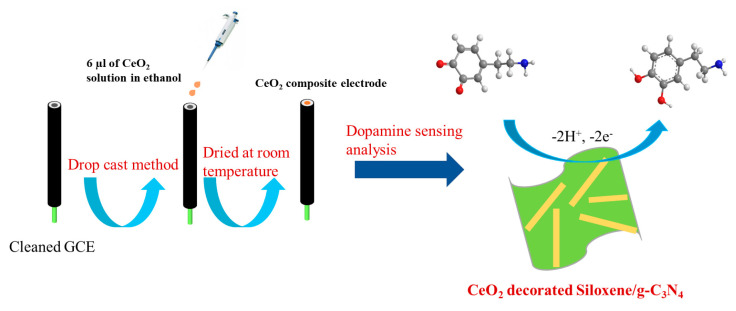
Electrode modification process and dopamine sensing mechanism of the modified electrode.

Initially, aluminum oxide powder of 0.05 μM was used to polish a bare glassy carbon electrode (GCE). After that, the electrode was sonicated for 5 min in the deionized water and dried at room temperature. Meanwhile, 5 mg of active material (CeO_2_, CeO_2_/siloxene, CeO_2_/g-C_3_N_4_) was inserted into 5 mL of ethanol solution and sonicated for 30 min. Later, 6 μL of the active material solution was coated on the polished GCE by the drop-casting method and allowed to dry at room temperature. Scheme 2 shows the fabrication of the CeO_2_/siloxene- and g-C_3_N_4_-modified GCE electrodes and the DA sensing mechanism. Cyclic Voltammetry (CV) was used to optimize the electrodes for enhanced redox performance of DA detection. After that, the differential pulse voltammetry (DPV) technique was employed to investigate the DA detection of the modified electrode (CeO_2_/siloxene/GCE) in the 0.1 M phosphate-buffered saline (PBS, pH = 7.0) solution under different concentrations of DA. The interference study in the presence of interfering compounds was carried out after washing the electrodes by the consecutive addition of various analytes to avoid the formation of contaminates. The repeatability test was carried out with a CeO_2_/siloxene/GCE electrode for five consecutive runs, and the stability of the CeO_2_/siloxene/GCE electrode was examined for 15 days in the presence of 1000 µL of 0.2 mM of DA-containing 0.1 M PBS solution by the DPV measurement.

## 3. Results and Discussion

### 3.1. Structural and Morphological Characterization

**Figure 1:**
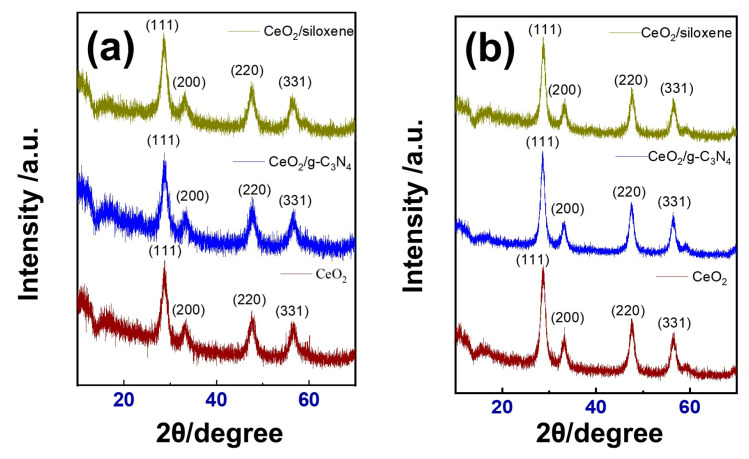
X-ray diffraction pattern of (**a**) cerium oxide (CeO_2_) and composites pyrolyzed at 350 °C, and (**b**) CeO_2_ and composites pyrolyzed at 450 °C.

The X-ray diffraction patterns of the Ce-MOFs composites and CeO_2_ composites are shown in Figure 1. The Ce-MOF’s sharp diffraction peaks are in close agreement with the XRD pattern of previously reported work [36], which confirms the better crystalline nature and phase purity of the Ce-MOF (Figure S1). After annealing, the diffraction peaks of the Ce-MOF were relocated at 28.7°, 33.1°, 47.4°, and 56.6°, corresponding to the (111), (200), (220), and (311) planes of CeO_2_ cubic phase, which matched with JCPDS card no. 34-0394 (Figure 1) [37]. The disappearance of the MOF peaks and the rise of new peaks after pyrolysis confirms the effective conversion of MOF to the crystalline phase of CeO_2_. The crystalline nature of CeO_2_ was investigated at two pyrolysis temperatures. As shown in Figure 1, the diffraction peak intensity of CeO_2_ prepared at 450 °C is higher than that of samples prepared at 350 °C, indicating better crystallinity increases in grain size. Interestingly, there is no distinct different peak among the CeO_2_, CeO_2_/siloxene, and CeO_2_/g-C_3_N_4_, probably due to the excellent crystallinity of CeO_2_ and the relatively low weight percentage of the siloxene and g-C_3_N_4_.

**Figure 2:**
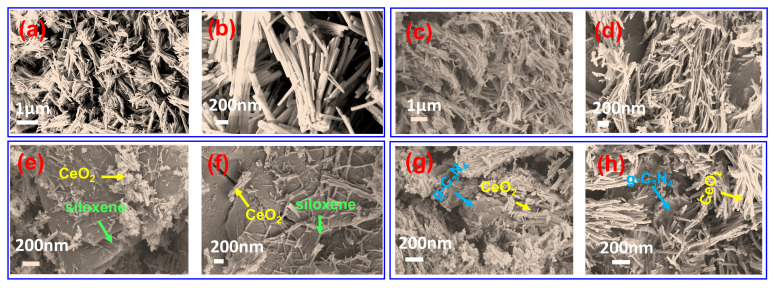
Scanning electron microscope (SEM) images of (**a**,**b**) Ce-MOF, pyrolyzed at 350 °C; (**c**) Bare CeO_2;_ (**e**) CeO_2_/siloxene; (**g**) CeO_2_/graphitic carbon nitride (g-C_3_N_4_), pyrolyzed at 450 °C; (**d**) Bare CeO_2;_ (**f**) CeO_2_/siloxene; and (**h**) CeO_2_/g-C_3_N_4_.

The morphologies of the samples as prepared were characterized by SEM and the images of the samples are shown in Figure 2. Figure 2a,b shows the nanorod structure of the Ce-MOF with a diameter of ~150 nm and lengths of a few micrometers. After being pyrolyzed, the obtained CeO_2_ retained a structure identical to that of the Ce-MOF without affecting the rod shape. Interestingly, a more extended nanorod structure was observed in the CeO_2_ sample prepared at 450 °C (Figure 2d), whereas some broken structures in the rod were observed for the CeO_2_ sample prepared at 350 °C (Figure 2c). The well-oriented nanorod CeO_2_ structure and its composites would enhance the redox abilities of DA sensors. Figure 2e–h shows the SEM images of CeO_2_/siloxene and CeO_2_/g-C_3_N_4_ samples, which were pyrolyzed at 350 °C and 450 °C, respectively. From these figures, the CeO_2_ nanorods were uniformly distributed on the surface of the siloxene and g-C_3_N_4_ with a slight agglomeration of CeO_2_ nanorods. Since the sensing properties of the active materials are strongly dependent on their crystalline nature and well-defined structure (as shown in Figure 1 and Figure 2), the samples pyrolyzed at 450 °C have been used for other characterization and electrochemical measurements, as a result of their higher crystallinity behavior.

**Figure 3:**
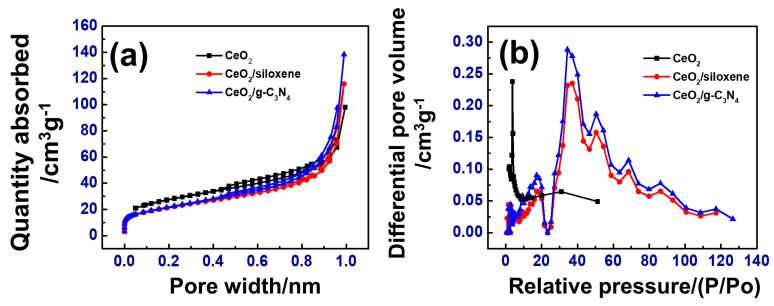
(**a**) Nitrogen adsorption–desorption isotherms; (**b**) Barrett–Joyner–Halenda (BJH) pore size distribution curves.

The surface area and porosity of the as-prepared CeO_2_-based composites (pyrolyzed at a temperature of 450 °C) were evaluated by BET analysis, and the N_2_ adsorption–desorption isotherms are shown in Figure 3a,b. As shown in Figure 3a, the isotherms of bare CeO_2_ and CeO_2_/siloxene and of CeO_2_/g-C_3_N_4_ showed the type IV isotherm having a sharp hysteresis loop in the relative pressure between 0.8 and 1.0, which suggests that the samples have a mesoporous structure. The BET surface area is calculated as 95.91, 75.73, and 78.23 m^2^ g^−1^ for CeO_2_, CeO_2_/siloxene, and CeO_2_/g-C_3_N_4_, respectively. The multilayer structure of siloxene and g-C_3_N_4_ might be responsible for dropping the surface area of the composites. Though the surface area of the CeO_2_/siloxene composite was lower than that of CeO_2_/g-C_3_N_4_, the planner structure of the siloxene is beneficial for π–π interaction with the dopamine structure, resulting in the enhanced sensing properties. Additionally, the mesoporous feature of the CeO_2_ composites was validated with a Barrett–Joyner–Halenda (BJH) analysis, as shown in Figure 3b. On the BJH curve, the samples exhibited a peak centered at around 3.7 nm, which could be the optimal pore size for electrolyte ion transportation during the electrochemical DA detection.

**Figure 4:**
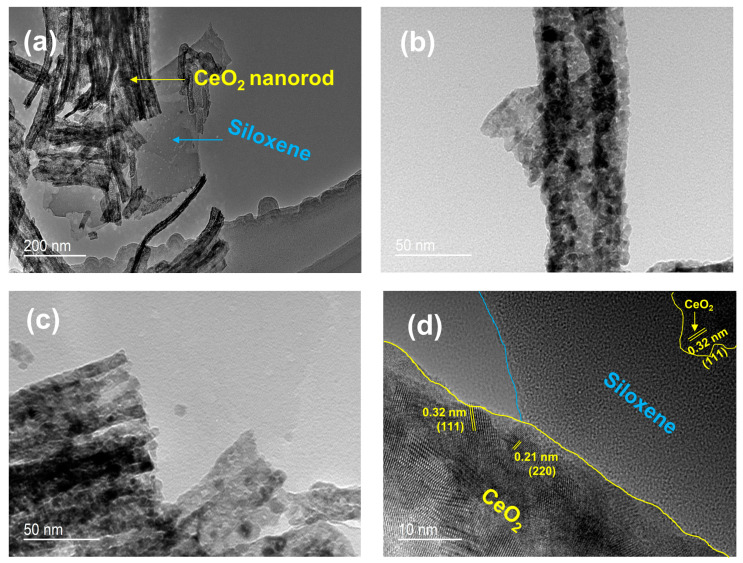
(**a**–**c**) Transmission electron microscopy (TEM) and (**d**) High-resolution transmission electron microscopy (HRTEM) images of the CeO_2_/siloxene composite.

**Figure 5:**
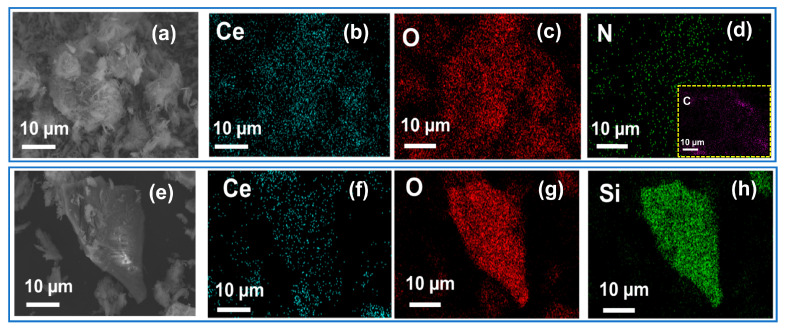
Energy-dispersive X-ray spectroscopy (EDS) elemental mapping of (**a**–**d**) CeO_2_/g-C_3_N_4_ and (**e**–**h**) CeO_2_/siloxene composites.

The morphology of CeO_2_/siloxene was further confirmed by high-resolution transmission electron microscopy (HRTEM) analysis. As seen in Figure 4a–d, CeO_2_ nanorods with uniform size (~50 nm) and lengths of a few micrometers could be observed in the CeO_2_/siloxene. Additionally, the siloxene sheets were firmly anchored with the CeO_2_ nanorods, which can enable faster electron transportation and active sites for DA oxidation. The HRTEM image validated the crystalline phase of CeO_2_ and the amorphous nature of the siloxene sheets (Figure 4d). Energy-dispersive X-ray spectroscopy (EDS) mapping was used to confirm the presence of the Ce, Si, and O elements in the CeO_2_/siloxene, and the presence of C, N, Ce, and O in the CeO_2_/g-C_3_N_4_. The obtained results are shown in Figure 5. As shown in Figure 5, the homogenous distribution of the elements can be found throughout the samples. Additionally, the CeO_2_/siloxene sample exhibited a high density of O and Si atoms distributed on the siloxene surface, which further confirms the strong coordination between the oxygen and silicon atoms in the siloxene.

### 3.2. Electrochemical Detection of Dopamine

**Figure 6:**
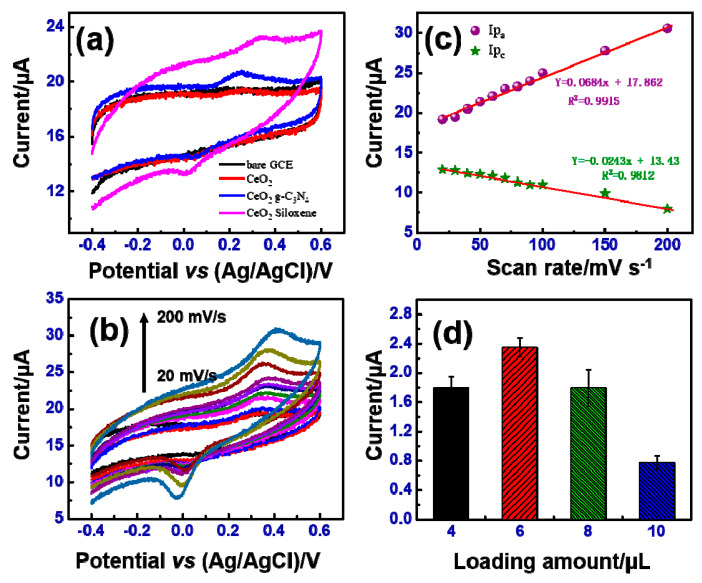
(**a**) Cyclic voltammetry response of bare glassy carbon electrodes (GCEs) and modified GCEs in 0.4 µM concentration dopamine (DA) containing 0.1 M phosphate-buffered saline (PBS) solution; (**b**) Cyclic voltammetry response of CeO_2_/siloxene-modified GCE electrode at different scan rates in 0.4 µM concentration DA containing 0.1 M PBS; (**c**) Calibration curve of the anodic and cathodic peak current versus the scan rate of the CeO_2_/siloxene-modified electrode; (**d**) The cyclic voltammetry peak current versus the different amounts of CeO_2_/siloxene loading.

Cyclic Voltammetry (CV) was employed to record the bare GCE and CeO_2_-, CeO_2_/g-C_3_N_4_-, and CeO_2_/siloxene-modified GCE electrodes’ electrochemical sensing performance. A pair of redox peaks were obtained for CeO_2_ and its composite-modified GCE electrodes in the presence of 0.4 µM of DA, which confirms the electrocatalytic activity of the modified electrodes for DA detection (Figure 6a). The redox peaks of the modified electrodes can be assigned to the oxidation of DA to dopaminequinone (DAQ) and the reduction of DAQ to DA [38]. The high CV current response was attributed to the larger surface area of the CeO_2_/siloxene/GCE electrode. It can be seen that the current response for DA oxidation is significantly enhanced for the CeO_2_/siloxene-modified electrode, which proves the outstanding catalytic behavior over other electrodes. The strong coordination modes between the Ce atom and siloxene oxygen functional group in CeO_2_/siloxene composite could improve the active sites for the DA oxidation process. From the CV results, the CeO_2_/siloxene-modified electrode was considered an optimal electrode for further electrochemical measurements towards DA detection.

The kinetics of the electrochemical process on the CeO_2_/siloxene-modified electrode surface was investigated by CV at different scan rates in the presence of 0.4 µM of DA containing 0.1 M of PBS electrolyte. As shown in Figure 6b, the oxidation and reduction peaks were shifted towards positive and negative directions upon increasing the scan rate from 20 mV s^−1^ to 200 mV s^−1^. Additionally, the anodic and cathodic (Ip_a_ and Ip_c_) peak currents showed an excellent linear relationship with the square root of the scan rate. They exhibited a correlation coefficient of 0.9915 and 0.9812, respectively (Figure 6c). This result confirms that DA’s oxidation behavior with the CeO_2_/siloxene/GCE electrode is an adsorption-controlled process [39].

Since the loading amount of the active materials on the electrode can influence the current response, the CV measurements for various amounts of CeO_2_/siloxene loaded on the GCE electrode were carried out in 0.4 µM of DA containing 0.1 M of PBS solution. As shown in Figure S2, the intensity of the redox peak and the current response of the 6 µL coated GCE electrode reached the maximum value, which confirms the higher DA oxidation activity on the electrode/electrolyte surface. After increasing the loading amount (8 µL and 10 µL), the redox peak current decreased gradually. As per Figure 6d, the GCE electrode coated with 6 µL of CeO_2_/siloxene material exhibited the highest DA oxidation current response, which indicates the significant electron transportation at the electrode/electrolyte interfaces. On the other hand, excessive loading of CeO_2_/siloxene may lead to the formation of a thicker film with aggregated CeO_2_/siloxene on the GCE surface, and could hinder the DA’s interaction with the electrode. Thus, electron transportation was blocked to the electrode surface due to the increase of the active material film’s thickness on the GCE, resulting in a lower DA oxidation current [40,41]. From this, 6 µL of CeO_2_/siloxene is considered to be the optimal loading for further electrochemical analysis.

### 3.3. DA Detection in the CeO_2_/Siloxene-Modified GCE Electrode

**Figure 7:**
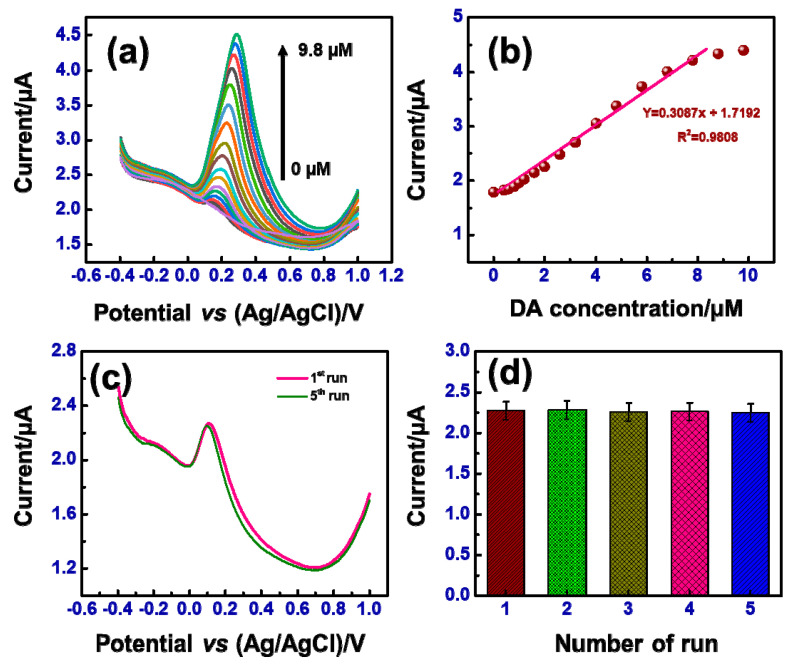
(**a**) Differential pulse voltammetry (DPV) response of the CeO_2_/siloxene-modified electrode in 0.1 M PBS solution containing different concentrations of DA; (**b**) Linear response of the oxidation current versus DA concentrations; (**c**) Repeatability of a modified electrode in the presence of 1 µM of DA-containing 0.1 M PBS solution; (**d**) Plot of the oxidation current versus the run number for five consecutive measurements.

The differential pulse voltammetry technique (DPV) has several advantages: operational simplicity, high sensitivity, high accuracy, and a broad linear range for DA detection [42]. Therefore, the DPV technique was used to recognize the dopamine sensing ability of the CeO_2_/siloxene-modified electrode under the optimized conditions. Figure 7a shows the DPV response of the CeO_2_/siloxene-modified electrode at various concentrations of DA containing 0.1 M of PBS solution. From Figure 7a, it can be seen that the DA oxidation current response of the electrode gradually increased with increasing concentrations of DA (0 to 9.8 µM), proving the excellent electrocatalytic behavior of the electrode for dopamine. The fabricated CeO_2_/siloxene sensor possesses a good linear range between DA concentration and the oxidation current with a correlation coefficient R^2^ = 0.9808 (Figure 7b). Moreover, the sensor exhibited a detection limit of 0.292 μM.

Table 1 shows the electrocatalytic DA sensing performance of our sensor compared with previously reported electrodes. As the table shows, the CeO_2_/siloxene sensor exhibited a superior performance of DA detection, which shows a promising application in the biosensor field. The repeatability of the sensor is an essential characteristic of the electrochemical detection of DA. Figure 7c shows the DPV current response of a CeO_2_/siloxene-modified electrode in 0.4 µM DA containing 0.1 M PBS solution for ten replicant measurements. The DA oxidation current response of all of the runs is almost at the same level, demonstrating the excellent repeatability of the sensing performance.

**Figure 8:**
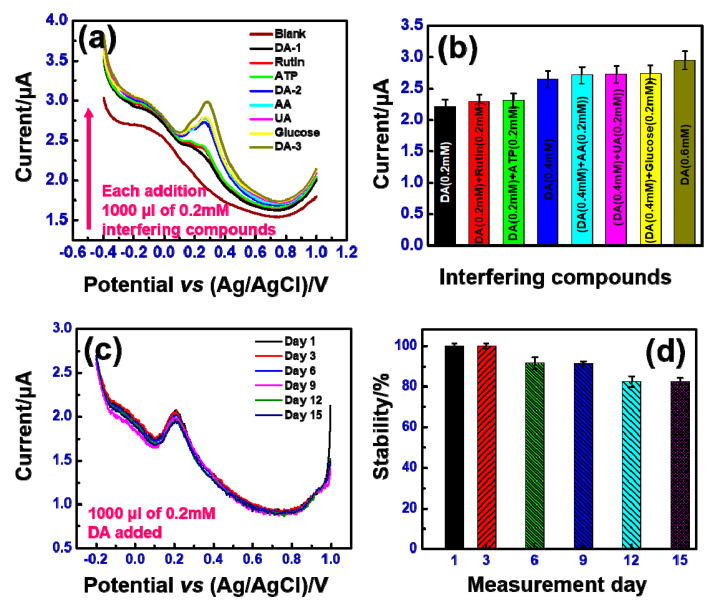
(**a**) Interference analysis of the CeO_2_/siloxene-modified electrode in the presence of other interfering compounds; (**b**) Current change response to the interfering compounds; (**c**,**d**) Stability of the CeO_2_/siloxene-modified electrode in the presence of 1000 µL of 0.2 mM of DA containing 0.1 M PBS solution.

**Figure 9:**
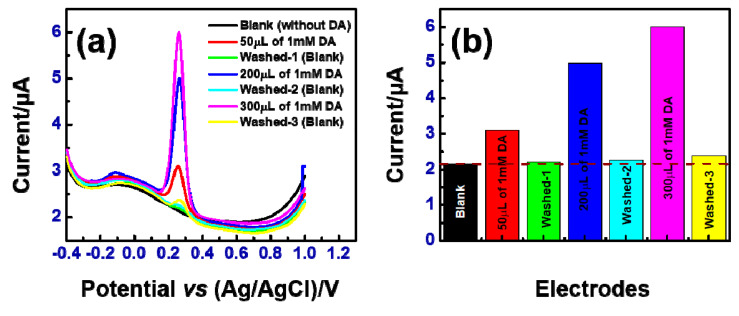
(**a**) DPV response of the CeO_2_/siloxene modified electrode before and after washing in blank and DA containing PBS solutions; (**b**) Current changes bar chart before and after washed the electrodes.

Selectivity is another crucial factor in discriminating between some common species in similar analytes. Figure 8 represents an interference study of the CeO_2_/siloxene-modified electrode in the presence of other interfering compounds such as ascorbic acid (AA), uric acid (UA), Adenosine triphosphate (ATP), glucose, and rutin. As Figure 8a shows, the initial current response was observed after the addition of 1000 µL of 0.2 mM DA solution into 0.1 M PBS electrolyte. Afterwards, 1000 µL of 0.2 mM of rutin and ATP were added, and negligible changes in the oxidation current could be noticed. A remarkable change in the oxidation current was observed after the second addition of 1000 µL DA, indicating the sensor’s excellent selectivity. Furthermore, the other interfering compounds (AA, UA, and glucose, 1000 µL of 0.2 mM) were also injected into the PBS solution, which revealed no fluctuations in the current and maintained the same DA oxidation current response (Figure 8b). The results prove that the CeO_2_/siloxene-modified electrode has better selectivity towards detecting DA molecules under optimal conditions. Stability is a vital characteristic for sensors; the DPV analysis was performed to investigate the stability of the CeO_2_/siloxene-modified GCE electrode. The electrode was kept in the refrigerator when not in use. As per Figure 8c, there was only a tiny change in the DA oxidation current even after 15 days compared with the initial measurement (first day). The DA oxidation current of 82.3% was maintained after two weeks of measurements, which indicates the good stability of the modified electrode for the DA sensor. Additionally, the sensor’s recovery was investigated by the DPV technique before and after washing the electrodes in the PBS solution and DA containing the PBS solution. As per Figure 9a,b, the CeO_2_/siloxene-modified electrode possessed an excellent recovery characteristic in the PBS solution after washing the electrodes. However, a tiny change in the current response was observed after washing the electrode (washed 2 and washed 3 in Figure 9b) compared with the initial measurement (blank) in the PBS solution, suggesting the formation of a small quantity of contaminants on the electrode surface. Although a small difference was noticed after washing the electrodes, it was negligible compared to the DA oxidation current. These results demonstrate that one-dimensional (1D) CeO_2_ nanorods with 2D siloxene sheets could be employed as a selective dopamine sensor.

## 4. Conclusions

In this work, we successfully combined Ce-MOF with siloxene and g-C_3_N_4_ and prepared CeO_2_/siloxene and CeO_2_/g-C_3_N_4_ composites using the pyrolysis method. The as-prepared samples were characterized by different tools and optimized the pyrolysis condition for better crystallization of CeO_2_. Compared with pure CeO_2_ and CeO_2_/g-C_3_N_4_-modified electrodes, the CeO_2_/siloxene-modified GCE exhibited superior electrochemical performance towards DA detection thanks to its strong redox mode between Ce and siloxene functional groups. The proposed sensor showed a detection limit of 0.292 μM with a linear range between 0.292 μM and 7.8 μM. Moreover, the CeO_2_/siloxene-modified sensor possessed good repeatability and selectivity for DA detection. This work provides a new insight into preparing CeO_2_ with siloxene from an MOF for highly electrocatalytic DA sensors and could be extended to the detection of other suitable biomolecules in the near future.

## Figures and Tables

**Table 1:** Comparison of the analytical properties of other reported dopamine (DA) electrochemical sensors.

Electrode	Technique	Limit of Detection (μM)	Linear Range (μM)	Ref.
Beansprout/(SAM)/Au	SWV	0.478	9.9–2210	[43]
Tyrosinase/NiO/ITO	CV	1.032	2–100	[44]
P(TBA_0.50_Th_0.50_)	EIS/CV	0.3	7.8–125	[45]
PA-MWCNT/GCE	EIS	14.1	10–1000	[46]
rGO/TiO_2_	DPV	1.5	1–35; 35–100	[47]
Ag/Graphene/GCE	LSV	5.4	10–800	[48]
**CeO_2_/siloxene/GCE**	**DPV**	**0.292**	**0.292–7.8**	**This work**

## Supplementary Materials

The following are available online at https://www.mdpi.com/1424-8220/20/17/4880/s1, Figure S1: XRD pattern of the Ce-MOF, Figure S2: Cyclic voltammetry response of different amount of CeO_2_/siloxene loading in 0.4 µM concentration DA containing 0.1 M PBS solution.
