# Supplementary material for: CeO2-Based Two-Dimensional Layered Nanocomposites Derived from a Metal–Organic Framework for Selective Electrochemical Dopamine Sensors
Source: Sensors (Basel). 2020 Aug 28;20(17):4880. doi: 10.3390/s20174880 (PMC7506630; doi:10.3390/s20174880)
Supplement: Supplementary file 1 [file sensors-20-04880-s001.pdf]

sensors

Supplemental information

CeO_2-Based Two-Dimensional Layered Nanocomposites Derived from a Metal–Organic Framework for Selective Electrochemical Dopamine Sensors

Chengjie Ge [1], Rajendran Ramachandran [1,2], and Fei Wang [1,3,*]

[1] School of Microelectronics, Southern University of Science and Technology, Shenzhen 518055, China; 11712522@mail.sustech.edu.cn
[2] SUSTech Academy for Advanced Interdisciplinary Studies, Southern University of Science and Technology, Shenzhen 518055, China; ramachandran@sustech.edu.cn
[3] Engineering Research Center of Integrated Circuits for Next-Generation Communications, Ministry of Education, Shenzhen 518055, China;
* Correspondence author: wangf@sustech.edu.cn

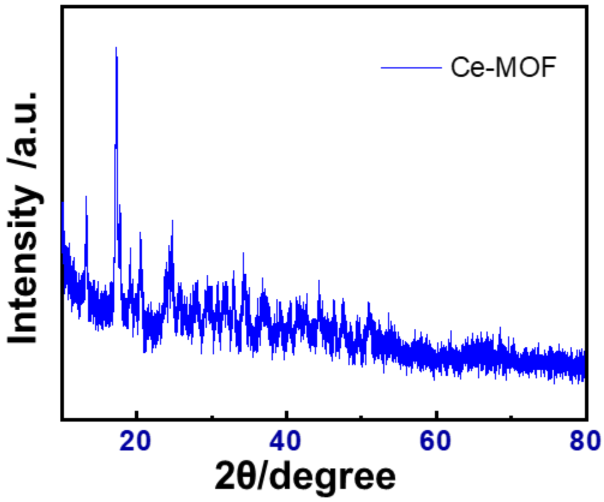

Figure 1. XRD pattern of the Ce-MOF.

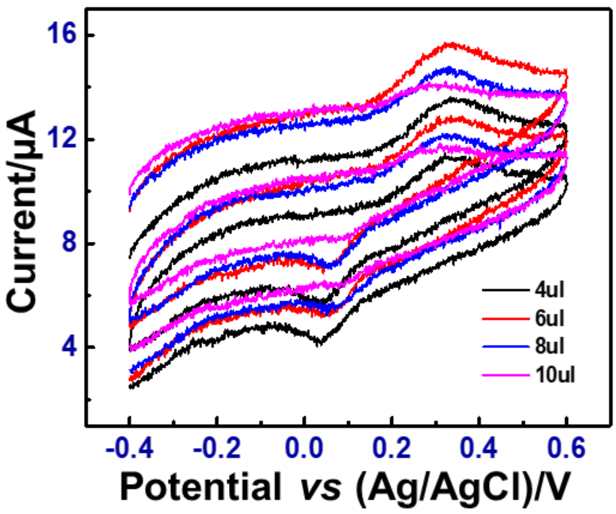

Figure 2. Cyclic voltammetry response of different amount of CeO_2/siloxene loading in 0.4 μM concentration DA containing 0.1M PBS solution.

© 2020 by the authors. Submitted for possible open access publication under the terms and conditions of the Creative Commons Attribution (CC BY) license (http://creativecommons.org/licenses/by/4.0/).
